# SDS Interferes with SaeS Signaling of *Staphylococcus aureus* Independently of SaePQ

**DOI:** 10.1371/journal.pone.0071644

**Published:** 2013-08-20

**Authors:** Phuti E. Makgotlho, Gabriella Marincola, Daniel Schäfer, Qian Liu, Taeok Bae, Tobias Geiger, Elizabeth Wasserman, Christiane Wolz, Wilma Ziebuhr, Bhanu Sinha

**Affiliations:** 1 Institute for Hygiene and Microbiology, University of Würzburg, Würzburg, Germany; 2 Institute for Medical Microbiology and Hygiene, University of Tübingen, Tübingen, Germany; 3 Department of Microbiology and Immunology, Indiana University School of Medicine-Northwest, Gary, Indiana, United States of America; 4 Department of Pathology, Stellenbosch University, Cape Town, South Africa; 5 Institute for Molecular Infection Biology, University of Würzburg, Würzburg, Germany; 6 Department of Medical Microbiology and Infection Prevention, University Medical Center Groningen, Groningen, The Netherlands; Institut Pasteur, France

## Abstract

The *Staphylococcus aureus* regulatory *saePQRS* system controls the expression of numerous virulence factors, including extracellular adherence protein (Eap), which amongst others facilitates invasion of host cells. The *saePQRS* operon codes for 4 proteins: the histidine kinase SaeS, the response regulator SaeR, the lipoprotein SaeP and the transmembrane protein SaeQ. *S. aureus* strain Newman has a single amino acid substitution in the transmembrane domain of SaeS (L18P) which results in constitutive kinase activity. SDS was shown to be one of the signals interfering with SaeS activity leading to inhibition of the *sae* target gene *eap* in strains with SaeS^L^ but causing activation in strains containing SaeS^P^. Here, we analyzed the possible involvement of the SaeP protein and *saePQ* region in SDS-mediated *sae*/*eap* expression. We found that SaePQ is not needed for SDS-mediated SaeS signaling. Furthermore, we could show that SaeS activity is closely linked to the expression of Eap and the capacity to invade host cells in a number of clinical isolates. This suggests that SaeS activity might be directly modulated by structurally non-complex environmental signals, as SDS, which possibly altering its kinase/phosphatase activity.

## Introduction

*Staphylococcus aureus* is part of the commensal flora, colonizing predominantly the anterior nares of approximately 20–50% of the human population [1]. However, it is also a facultative pathogen able to cause a wide spectrum of infections, ranging from skin and soft tissue infections and abscess formation to complicated systemic diseases such as osteomyelitis, endocarditis, sepsis and toxic shock syndrome [2], [3]. *S. aureus* has the ability to rapidly adapt to different environmental conditions, including heat, pH, and a range of chemical components. There is now growing evidence that *S. aureus* can also invade and persist within different cell types. The invasion potential is due to the production of various proteins such as fibronectin binding proteins (FnBPs) and extracellular adhesive proteins (Eap) [4] which are controlled by the regulatory SaePQRS system [5]. Strain Newman uses Eap rather than FnBPs as invasin since both FnBPs are secreted due to a point mutation resulting in a truncation of these proteins [6].

SaeR and SaeS are part of a bacterial two-component system coding for a response regulator and a histidine kinase, respectively [7]. They are encoded in the *saePQRS* operon together with other two ORFs, which are predicted to encode a lipoprotein (SaeP) and a membrane protein (SaeQ). Recently it was suggested that these two proteins play a role in the deactivation of the the *sae* system by inducing the phosphatase activity of SaeS [8], [9].

**Figure 1:**
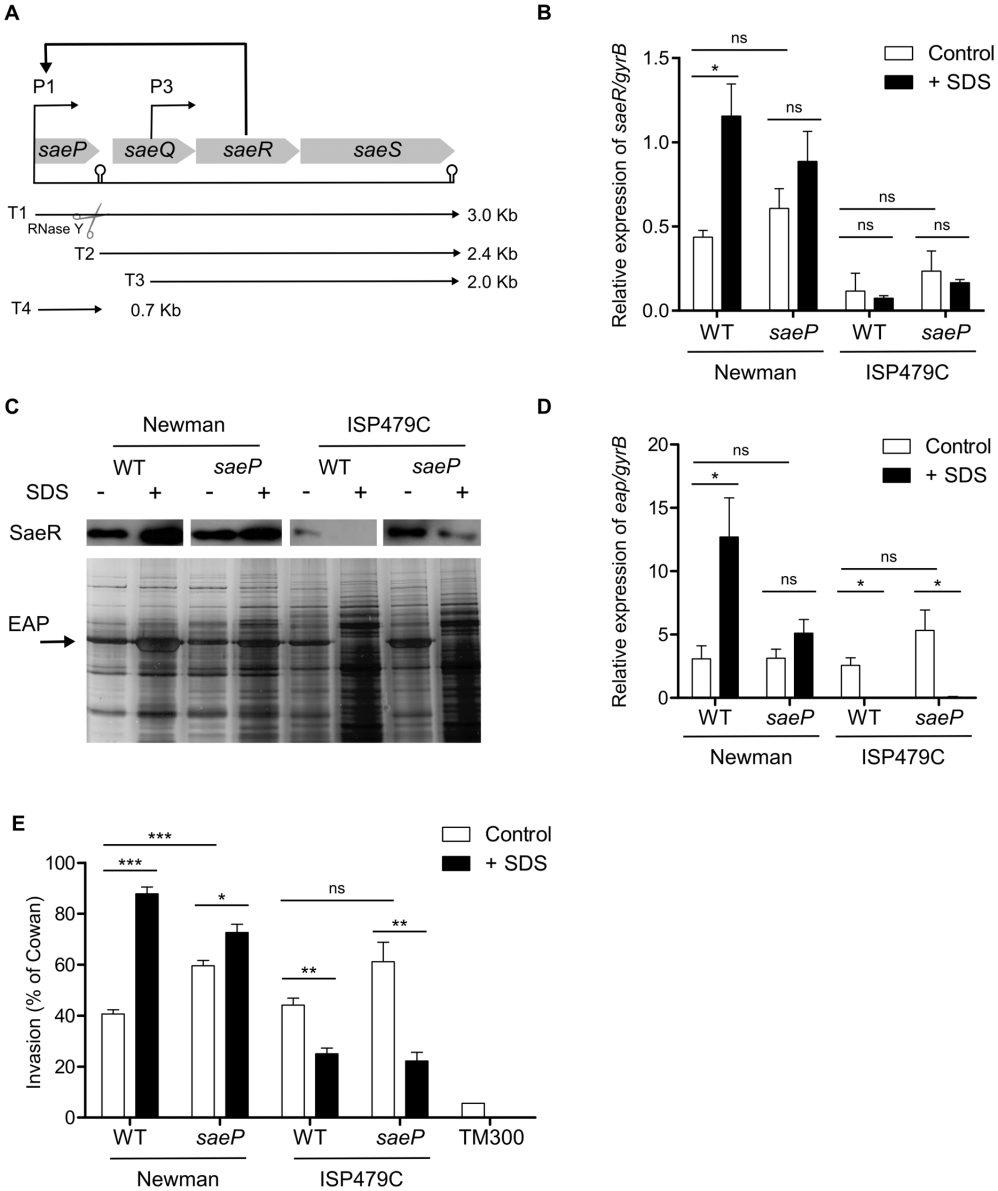
*saeP* deletion does not affect SDS-mediated *sae* activity. (**A**) Schematic representation of the *sae* locus with its four ORFs. Two promoters, P1 and P3 generate three primary transcripts (T1, T3, and T4). T1 processing by an endonucleolytic enzyme, RNase Y, results in T2. (**B, C, D, E**) Wild type and *saeP*-deleted strain in Newman and ISP479C backgrounds were grown in TSB without (-) or with (+) SDS (30% MIC) until late exponential growth phase. (**B**) Relative expression of *saeR* in relation to *gyrB* was assessed by qRT–PCR. The results represent means ± SEM of at least three independent experiments performed in triplicates. (**C**) (**Lower panel**) Expression of Eap was monitored by SDS PAGE and silver staining. (**Upper panel**) Expression of SaeR was monitored by Western blot analyses with specific antibody against SaeR. (**D**) Relative expression of *eap* in relation to *gyrB* was assessed by qRT–PCR. The results represent means ± SEM of at least three independent experiments performed in triplicates (**E**) Cellular invasion of 293 cells was measured and expressed as relative invasiveness compared to *S. aureus* strain Cowan I. Results represent means ± SEM of at least three independent experiments performed in duplicates. (**B,D,E**) Asterisks indicate the significance of comparisons (***P<0.001; **P = 0.001–0.01; *P = 0.01–0.05; ns P>0.05).

A total of four overlapping transcripts (T1–T4) are expressed in the *sae* operon from two promoters (P1 and P3) (Fig. 1A) [10]. The T1 transcript is transcribed from the strongly auto-activated P1 promoter [10]. The most abundant and stable T2 transcript is generated by endoribonucleolytic cleavage of T1 by RNase Y [10], [11]. T3 is transcribed from the weak constitutive P3 promoter [10] and, finally, T4 is a monocistronic transcript coding just for *saeP* [12], [13].

The *sae* system can be activated by environmental stimuli such as, H_2_O_2_, low pH, and sub-inhibitory concentrations of α-defensins and antibiotics [10], [14]–[16]. We could show previously that sub-inhibitory concentrations of sodium dodecyl sulfate (SDS) lead to a decrease of *sae* target gene expression (e.g. *eap*) in some *S. aureus* strains, but causes an increase in strain Newman [17]. This opposing effect was mirrored by a decrease and increase of the invasion capacity of the strains upon SDS treatment, respectively.

Strain Newman is characterized by a high, constitutive expression of the *sae* operon due to an amino acid substitution (Proline for Leucine, L18P) within the putative N-terminal transmembrane domain of the sensor histidine kinase SaeS (SaeS^P^). Several lines of evidence led to the conclusion that the SaeS^P^ allele renders the kinase constitutively active [5], [9], [10], [12]. Thus the Sae system of strain Newman is thought to be non-responsive to environmental signals. Of note, SDS is the only signal described so far which seems to activate the SaeS of strain Newman.

Here we analyze the possible involvement of the upstream part of the *sae* operon (i.e. *saePQ*) in SDS-mediated *sae* activation. We could show that the upstream region does not interfere with SDS signaling in any of the strains analyzed (i.e. carrying either SaeS^P^ or SaeS^L^). Moreover, we could show that the auto-regulated promoter P1 is also dispensable in SDS-mediated *sae* activation.

## Materials and Methods

### Bacterial Strains and Growth Conditions

Strains and plasmids used in this study are listed in Table 1. *S. aureus* strains were grown in TSB medium. For strains carrying resistance genes, antibiotics were used only in overnight cultures at the following concentrations: 10 µg ml^−1^ erythromycin and 5 µg ml^−1^ tetracycline. Bacteria from overnight cultures were diluted in fresh TSB to an initial optical density at 600 nm (OD_600_) of 0.05 with and without SDS 0.004% (w/v), and grown with shaking at 200 rpm at 37°C to the desired growth phase. SDS MIC of the strains used in this study was determined by microdilution (not shown). The concentration of SDS was chosen at 30% of the MIC.

**Table 1:** Bacterial strains and plasmids.

Strain or plasmid	Description	Reference
Strains		
* E. coli*		
TOP10	Competent *E. coli* for plasmid transformation	Invitrogen
* S. aureus*		
RN4220	Restriction-deficient *S. aureus* strain, r^−^	[23]
Newman	Wild type	[24]
Newman-29	Newman *sae::kan*	[10]
Newman-31	Newman *saeP::kan*	This study
NewmanHG	Newman, with SaeS^L^ from strain RN1	[5]
ISP479C	8325-4 derivative, with SaeS^L^ allele	[25]
ISP479C-29	ISP479C *sae::kan*	[10]
ISP479C-31	Newman *saeP::kan*	This study
6850	Wild type	[26]
ATCC29213	MSSA reference strain	This study
LAC	CA-MRSA (USA300)	[27]
MW2	CA-MRSA (USA400)	[28]
ST239-635/93 W	MRSA SCCmec type III reference strain	Provided by F. Layer (Robert Koch Inst. Wernigerode, Germany
ST239-635/93 R	MRSA SCCmec type III strain	This study (spontaneous mutant from ST239-635/93 W)
ST239 (THW89)	MRSA SCCmec type III isolated from skin & softtissue infection	Provided by W. Oosthuysen (Tygerberg Hospital, South Africa)
ST239 (THW99)	MRSA SCCmec type III isolated prostheticdevice associated infection	Provided by W. Oosthuysen (Tygerberg Hospital, South Africa)
ST239 (465)	Zoonotic isolate	Provided by Knut Ohlsen (University of Würzburg, Germany)
ST2393 (966)	Zoonotic isolate	Provided by Knut Ohlsen (University of Würzburg, Germany)
Cowan I	Wild type	ATCC 12598
* S. carnosus*		
TM300	Wild type	[29]
Plasmids		
pMAD	Vector for allelic replacement	[19]
PCWSAE31	pMAD with cloned *saeP::kan*	This study
PCWSAE28	pCL84 with *saePQRS* from ISP479C	[5]
PCWSAE33	pCL84 with *saePQRS* from Newman	[5]
PCWSAE42	pCL84 with *saeRS* from Newman	(T. Geiger, unpublished)
PCWSAE47	pCL84 with *saeRS* from ISP479C	[10]

### Construction of the saeP Mutant

The *saeP* locus was replaced by a kanamycin resistant cassette. Briefly, two fragments flanking *saeP* and the *KanA* gene were amplified and annealed by overlapping PCR using the oligonucleotides listed in Table 2. The amplicon was restricted with KpnI and cloned into pBT2 [18]. To take advantage of blue-white selection, the fusion fragments were then sub-cloned into the EcoRI and SalI sites of pMAD [19], yielding plasmid pCWSAE31. This plasmid was then verified and transformed into RN4220, where mutagenesis was performed as described previously [20]. The mutant (referred to as RN4220-31) was verified by PCR and pulsed-field gel electrophoresis. After mutagenesis, the resulting mutation was transduced into the experimental strains.

**Table 2:** Oligonucleotides.

Purpose	Template	Name	Sequence
**Mutagenesis**			
* saeP* replacementmutant			
	ISP479C	Kpnsae-for	CGGGGTACCATACTACAGTTTTACATT
		Kpn-ORF4-rev	CACCTCGGTACCCTGTTCTTACGACCTCTAAAG
	ISP479C	HybridORF4a-rechts	TAAAAGTTCGCTAGATAGGGGTCCCCTTCC TGTTCACATAACA
		Hybridsae-links	TCCAATTCTCGTTTTCATACCTCGGAGCTAA CTCCTCATTTCTTCAATTT
	Newman-29	kanR-for	CCGAGGTATGAAAACGAGAATTGG
		kanR-rev	GGGACCCCTATCTAGCGAACTTT
**DIG DNA** **probes**			
* sae*	RN6390	sae1980-for	TGGTCACGAAGTCCCTATGC
		sae2458-rev	TGCTTGCGTAATTTCCGTTAG
* eap*	RN6390	Map w 98	AATAATAATGAAGCGTCTGC
		Map w 650	CGGTAATACCTCTATTTGATT
		ribD-dig-rev	CAAAGTTCCAACTCCTCTTTTA
**qRT-PCR**			
* saeR*		saeRS-up	AAACTTGCTTGATAATGCGCTAAA
		saeRS-dw	TTCTGGTATAATGCCAATACCTTCA
* gyr*		gyrB-up	TTAGTGTGGGAAATTGTCGA
		gyrB-dw	CCGCCGAATTTACCACCAGC
* eap*		eap-up	AAGCGTCTGCCGCAGCTA
		eap-dw	TGCATATGGAACATGGACTTTAGAA

### Sodium Dodecyl Sulphate-polyacrylamide Gel Electrophoresis (SDS-PAGE)

For surface protein profile analysis, 5 ml of *S. aureus* culture grown to the desired growth phase in 50 ml of TSB was pelleted and washed with 5 ml of PBS. Subsequently, 1 ml of the washed culture was pelleted and suspended in 40 µl of Laemmli’s buffer. The suspension was boiled at 100°C for 15 min and subsequently centrifuged at 5000 rpm for 5 min. 8 µl of the supernatant was loaded onto a 12% polyacrylamide gel and run at 100 V for 2 hours. After electrophoresis, proteins were stained with silver nitrate (Merck, Germany) and analyzed by a GS800 calibrated densitometer. Bands were assigned to proteins according to their migration, as previously identified by liquid chromatography-tandem mass spectrophotometry [17]. For Western blot analysis, proteins from SDS-PAGE were transferred to a nitrocellulose membrane (Watman, Dassel, Germany) using the Mini Transblot Cell system (Biorad). The membrane was blocked with blocking solution (5% skim milk and 1% Tween) for 1 h. Subsequently, nitrocellulose membranes were incubated overnight at 4°C with diluted anti-SaeR primary antibody (1∶2000). The secondary antibody was an anti-rabbit immunoglobulin G horseradish peroxidase-conjugated (Jackson Immunoresearch, Germany). Detection was done with an ECL kit (GE Healthcare, UK) following the manufacturer’s instructions.

### RNA Isolation and Northern Blot Hybridization

Total RNA from *S. aureus* cultures was isolated and purified by the Trizol method or RNeasy Mini Kit (Qiagen, Hilden, Germany) without any detectable differences. RNA extraction using the RNeasy Mini kit was performed according to the manufacturer’s instructions. Briefly, 2 ml of *S. aureus* culture was pelleted by centrifugation at 13 000 rpm for 5 min. Subsequently, the pellet was washed with 1 ml of PBS and centrifuged at 13 000 rpm for 5 min. After the washing step, the pellet was resuspended in 700 µl RLT buffer. The suspension was transferred to 0.1 mm silica spheres lysing matrix tubes (MP Biomedicals Ohio, USA) and lysed by mechanical disruption in with the Fastprep-24 (MP Biomedicals Ohio, USA) at 6500 rpm for 45 sec. Following disruption of the bacterial cells, the supernatant was transferred to a 2 ml tube and centrifuged at 13 000 rpm for 10 min at 4°C. The supernatant was transferred to a new 1.5 ml tube and equilibrated with 70% ethanol. The mixture was then transferred to an RNeasy column (Qiagen, Hilden, Germany) and from this step on the manufacturer’s instructions were followed. For the trizol method, RNA isolation was performed as described previously [21]. Briefly, bacteria were lysed in 1 ml of Trizol reagent (Invitrogen) with 0.5 ml of zirconia-silica beads (0.1-mm diameter) in a high-speed homogeniser (Savant Instruments, Farming-dale, NY). RNA was then isolated as described by the Trizol manufacturer’s protocol.

Northern blot analysis was performed as previously described [21]. Digoxigenin (DIG)-labelled DNA probes for the detection of specific transcripts were generated with a DIG-labelling PCR kit as described by the manufacturer’s instructions (Roche Biochemicals) using the oligonucleotides listed in Table 2. Detection was performed by a chemiluminescence kit (Roche, Mannheim, Germany) and subsequent exposure of X-ray films.

### Quantitative Real-time RT-PCR Analysis

For quantitative RT–PCR (qRT–PCR), 1 µg of total mRNA was reverse transcribed into cDNA using the Omniscript RT-PCR kit (Qiagen; Hilden, Germany) following the manufacturer’s protocol. cDNA was used for qRT-PCR analysis using the SYBR Green PCR Master Mix (Applied Biosystems; Warrington, UK). Thermal cycling, amplification and detection were performed with the StepOnePlusTM Real-Time PCR system (Applied Biosystems; Warrington, UK). Transcript abundance was calculated by the ΔΔCT method [22] using a dilution series of Newman wild type RNA as a standard. Subsequently, relative quantification was calculated in relation to the reference gene *gyrB*. Means were calculated from at least two biological replicates run in triplicates. Data were analyzed with 7300 Fast System Software (Applied Biosystems, Warrington, UK).

### Flow Cytometric Invasion Assay

Cellular invasiveness of *S. aureus* strains to 293 cells (human embryonic kidney, also referred to as HEK293 cells) was determined as previously described with minor modifications [17]. Briefly, 5 ml of *S. aureus* culture in 50 ml TSB with/out SDS as previously described was pelleted and washed with PBS. Subsequently to washing, bacteria were harvested by centrifugation at 4000 rpm for 5 min at 4°C. The bacteria were labeled with 3 ml of fluorescein isothiocyanate (FITC 1 mg/ml in 10% dimethyl sulfoxide) for 30 min at 37°C. The bacteria were then washed with 5 ml of PBS and centrifuged at 4000 rpm for 5 min. After centrifugation, the pellet was suspended in 1% human serum albumin-PBS (HSA)-PBS to a final OD_540_ of 1. For the bacterial invasion assay, the 293 cells (3×10^5^ cells/well) were plated one day before the experiment. Right before the assay, the cells were washed with invasion medium (1% HSA; 10 mM HEPES) (Dulbecco’s Modified Eagle’s Medium; Life Technologies, Carlsbad, CA) and, subsequently, 0.5 ml of invasion medium was added. Then, 50 µl suspension of FITC-labeled bacteria normalised to OD1_540 nm_ were added to the cells and sedimentation was allowed for 1 h at 4°C. Following sedimentation, culture plates were incubated at 37°C for 3 h with 5% CO_2_. Cells were then washed with 1 ml PBS and treated with monensin (25 µM final, dissolved in absolute ethanol) for neutralization in order to avoid fluorescence quenching by acidic pH. Propidium iodide (PI) was added to the cells in order to differentiate between live and dead cells, and cells were analyzed on a FACSCalibur (BD; California, USA). For the measurement of invasion, a forward scatter and side scatter (FSC/SSC) gating strategy was used. Florescence of the FITC+ cells was detected in the Fl-1 channel. In control experiment of uninfected 293 cells, less than 2% of PI-positive FSC/SSC-gated cells were detected in Fl-3 channel. Thus, experiments were run with the inclusion of PI positive cells; however, these were excluded in the final analyses.

Cellular invasiveness of *S. aureus* strains was determined as percentage relative of invasiveness of the *S. aureus* reference strain Cowan I and using *Staphylococcus carnosus* TM300 as negative control, as described previously [17].

### Statistical Analysis

Quantitative results are presented as standard error of means (SEM). Statistical analysis was performed using the Student’s two-tailed t-test unpaired. A P value of <0.05 was assumed as significant.

## Results and Discussion

### Influence of Saep on SDS-mediated Sae Activity

To determine whether SaeP is involved in the *sae* mediated response to sub-inhibitory concentration of SDS, *sae*P deletion mutants were generated in different *S. aureus* strains. Previously, we have shown that the point mutation in the sensor histidine kinase *SaeS* characteristic of strain Newman alters the response to SDS [17]. Therefore, *saeP* deleted mutants were generated both in strains Newman (carrying the SaeS^P^ allele) and in strain ISP479C (carrying the SaeS^L^ allele).

SDS response of the wild types and *saeP* mutants was monitored with different methods. *saeR* and SaeR expression were quantified by qRT-PCR and Western blot analyses (Fig. 1B and Fig. 1C: upper panel) respectively. *eap* and Eap expression were monitored qRT-PCR (Fig. 1D) and by SDS PAGE (Fig. 1C: lower panel), respectively. Moreover, since Eap is known to mediate *S.aureus* invasion of eukaryotic host cells [4], cellular invasion assays were performed (Fig. 1E).

As expected, SDS treatment resulted in a significant activation of *saeR* expression in strain Newman but slight (non-significant) reduction of *saeR* in strain ISP479C. Interestingly, deletion of *saeP* did not prevent induction or repression of *saeR* in either strain Newman or ISP479C (Fig. 1B). SaeR protein detection by Western blot analysis mirrored the *eap* transcription data and confirmed the diminished SaeR production upon SDS stress in strain ISP479C (Fig. 1C: upper panel). As expected, SDS treatment resulted in significant increase in *eap* expression in strain Newman [17] but repression in strain ISP479C (Fig. 1C, lower pane and Fig. 1D). Deletion of *saeP* did not alter this pattern of regulation (i.e. increase *eap* expression in strain Newman and decrease *eap* expression in strain ISP479C). Further on, the Newman *saeP* and ISP479C *saeP* mutants were tested for cellular invasiveness to 293 cells. The invasion data mirrored the *eap* expression (Fig. 1E). All these data taken together suggest that SaeP is not required for *sae*-mediated response to SDS.

Interestingly, *saeP* deletion led to an increased cellular invasiveness of Newman background. This last observation is in agreement with a recent study where SaeP together with SaeQ was shown to act as a suppressor of SaeRS mediated signaling [8].

### Saepq is not Needed for Sae-mediated Response to SDS

The data presented above strongly suggest that SaeP is not needed for *sae*-mediated response to SDS. To further analyze whether SaeQ or the P1 promoter interferes with SDS sensing, we studied the *sae*-mediated response to SDS in strains in which only *saeRS* was expressed from its native, constitutive P3 promoter, whose activity was previously shown to be independent of SaeR [10]. Constructs containing *saeRS^L^* or *saeRS^P^* were integrated into the chromosome of *sae* deletion strains. As controls, Newman wild type, and *saePQRS* deleted strains complemented with an integrated copy of the whole operon from the strain Newman (*saePQRS^P^*) or from strain ISP479C (*saePQRS^L^*) were included in the analyses.

**Figure 2:**
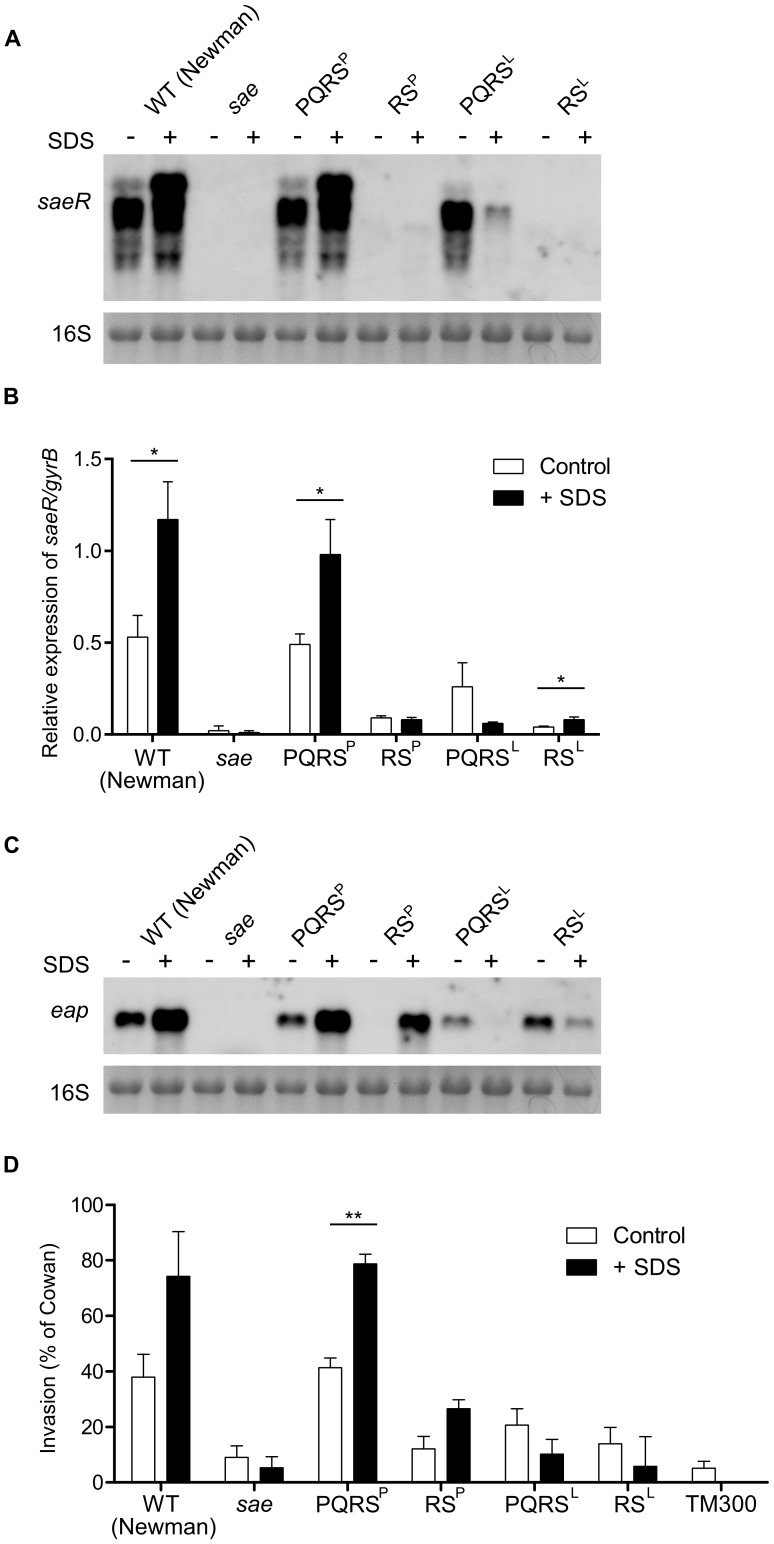
*saePQ* is not needed for *sae* mediated response to SDS. Wild type (Newman), *sae*-deleted, and *sae*-deleted strains complemented with *saePQRS^P^*, *saeRS^P^*, *saePQRS^L^*, *and saeRS^L^* were grown in TSB with or without SDS (30% MIC) until late exponential phase. (**A**) RNA was hybridized with a digoxigenin-labelled *saeR*-specific probe. 16S rRNA detected in ethidium bromide-stained gels is shown as a loading control. (**B**) Relative expression of *saeR* in relation to *gyrB* assessed by qRT–PCR. Results represent means ± SEM of at least three independent experiments performed in triplicates. (**C**) RNA was hybridized with a digoxigenin-labeled *eap*-specific probe. (**D**) Cellular invasiveness was measured in 293 cells and expressed as relative invasiveness compared to *S. aureus* strain Cowan I. Results represent means ± SEM of at least three independent experiments performed in duplicates. (**B,D**) Asterisks indicate the significance of comparisons (**P = 0.001–0.01; *P = 0.01–0.05).

Modulation of *sae* expression by SDS was analyzed using Northern blot hybridization with *saeR*-specific probes (Fig. 2A). As expected, SDS treatment led to an increase in *sae* transcription in Newman wild type (Fig. 2A, lane 2) [17]. The same induction was observed in a *sae*-deleted strain complemented with *saePQRS^P^*, thus indicating that the plasmid system used did not interfere with the analyses. In accordance, the *sae*-deleted strain complemented with *saePQRS^L^* responded to SDS in the same way that ISP479C (*i.e.* a decrease in *saeR* levels) (Compare Fig. 1B and Fig. 2A).

In the *sae*-deleted strains complemented with *saeRS^P^* or *saeRS^L^*, a very weak expression of *saeR* was observed by Northern blot. In order to quantify *saeR* expression in these strains, qRT-PCR was performed. As indicated in Fig. 2B, *saeR* levels in both the strain complemented with *saeRS^P^* and *saeRS^L^* was detectable at a similarly low level consistent with the weak activity from the P3 promoter. Moreover, the *saeRS* only constructs showed no altered expression in response to SDS exposure. This is in accordance with the fact that *saeRS* expression in these strains is exclusively dependent on the P3 promoter and therefore not subjected to P1 auto-regulation.

Next, the impact of the lack of *saePQ* on the modulation of *eap* expression by SDS was monitored by Northern blot analyses with the use of a specific probe against *eap* (Fig. 2C) and by cellular invasion assays (Fig. 2D).

An SDS modulation of *eap* expression was observed in all strains analyzed except for the *sae* deletion mutant. As expected, SDS treatment led to increase or decrease of *eap* expression in the *saePQRS^P^* and in the *saePQRS^L^* complemented strain, respectively (Fig. 2C). Interestingly, *eap* expression in the strains complemented with *saeRS^P^* or with *saeRS^L^* is still responsive to SDS stress (Fig. 2C), despite very low and SDS independent *saeRS* expression (Fig. 2B). This is in accordance to previous results showing that target gene expression is mostly dependent on SaeS activity with a minor impact of the SaeRS concentration [5], [9]. These data were corroborated by the cellular invasion assay (Fig. 2D) which strongly correlates with *eap* expression.

From these results we can speculate that SDS might possibly interact with the transmembrane part of SaeS protein either directly or through membrane perturbation. In strains harboring the native SaeS^L^ allele, SDS presumably leads to conformational changes resulting in a shift from kinase to the phosphatase activity. Lately, phosphatase activity was proposed to be enhanced by interaction with SaePQ [8]. However, SDS seems to control this switch independently of SaePQ. Of note, in strain Newman, harboring the SaeS^P^ allele, SDS has the opposite effect leading to a further activation of the already hyper-activated SaeS. This could be due to a further increase in kinase activity or alternatively to an inhibition of the low residual phosphatase activity in this strain. However, more experimental work is needed to substantiate this hypothesis.

### SDS Stress Affects *S. aureus sae* Expression in a Strain-dependent Manner

**Figure 3:**
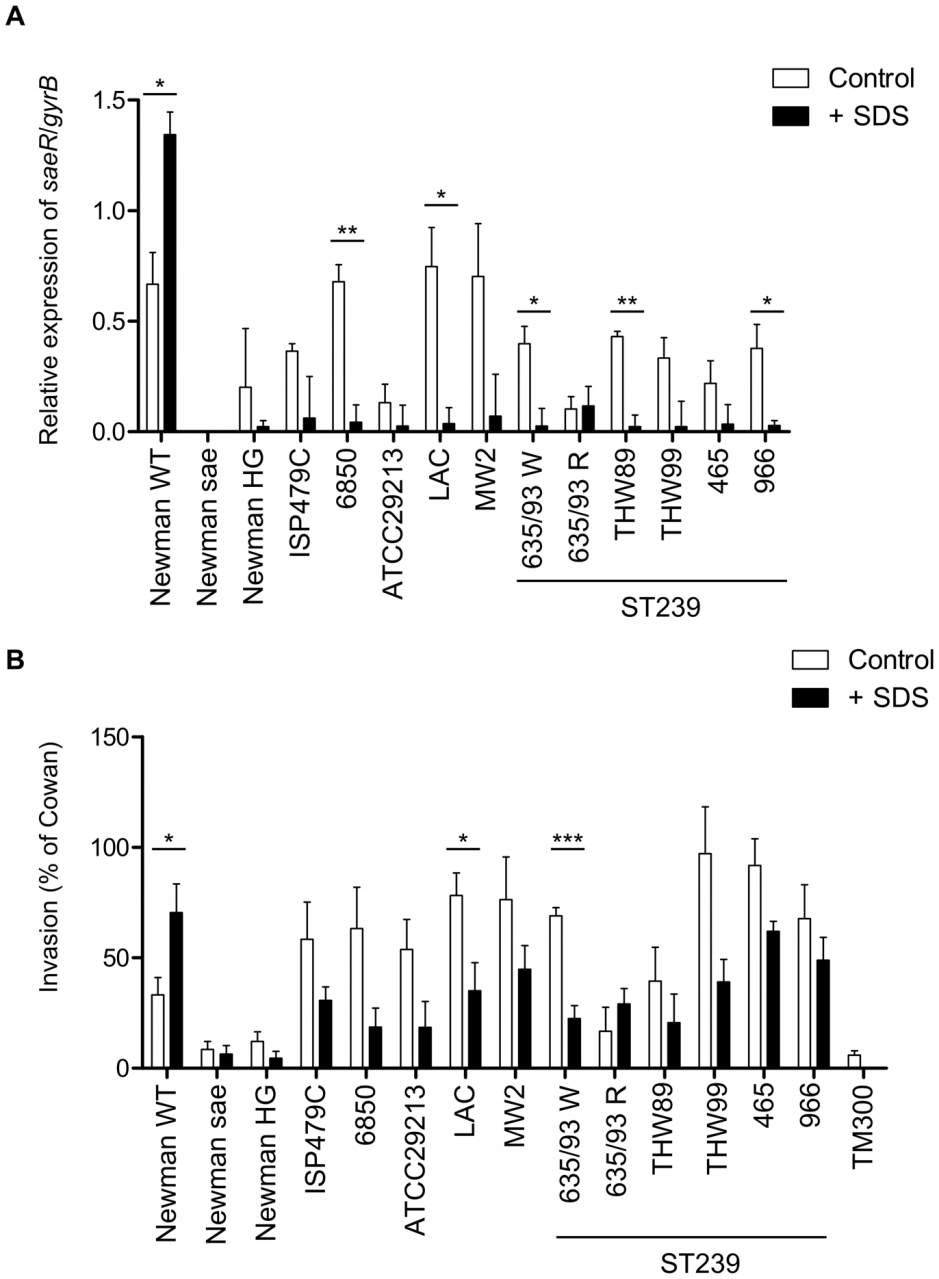
SDS stress affects *S. aureus sae* signaling in a strain-dependent manner. (**A**) Relative expression of *saeR* in relation to *gyrB* was assessed by qRT–PCR in various clinical isolates and in control strains at late exponential phase of growth. Results represent means ± SEM of at least three independent experiments performed in triplicates. (**B**) Cellular invasiveness into 293 cells was measured and expressed as relative invasiveness compared to *S. aureus* strain Cowan I. Results represent means ± SEM of at least three independent experiments performed in duplicates. (**A,B**) Asterisks indicate the significance of comparisons (***P<0.001; **P = 0.001–0.01; *P = 0.01–0.05).

Until now, the only strain which showed an up-regulation of the *sae* response by SDS was strain Newman [17]. We decided then to monitor the SDS response in various clinical strains by specific *saeR* qRT-PCR and the cellular invasion assay (Fig. 3). As controls, Newman wild type, an isogenic s*aePQRS* deletion mutant and strain Newman HG (where the *saeS* point mutation is reverted to the wild type [5]) were included in the analyses. With one exception, treatment with SDS resulted in inhibition of *sae* expression in all clinical strains analyzed (Fig. 3A). In the isolate ST239-635/93R *saeR* expression was low and no significant effect by SDS was observed (Fig. 3A). The sequence of the whole *saePQRS* operon was identical to those of strain 8325 and USA300 FPR3757 available from public databases. Interestingly, isolates which are closely related to ST239-335/93R all responded to SDS with down-regulation of *saeR* (Fig. 3A). Thus, the isolate ST239-635/93R seems to be unique with regard to the SDS response although no mutations in *saeS* or *saePQR* were detectable (data not shown).

Finally we analyzed whether SDS has a similar effect on the cellular invasion in clinical isolates (Fig. 3B). With one exception, treatment with SDS resulted in inhibition of cellular invasiveness capacities in all clinical strains analyzed (Fig. 3B). The isolate ST239-635/93R had a different (although not significant) response to SDS, namely enhance invasiveness to 293 cells by SDS. The mechanism by which SDS is able to enhance invasiveness of this strain remains to be elucidated.

It is interesting to note that strain Newman is not only peculiar with regard to SaeS but it is also characterized by non-functional FnBPs. Thus, in this strain invasion is only triggered by Eap. In the other strains analyzed, FnBPs are presumably the major invasins which play a role. Thus, the SDS effect on invasion may also be due to SDS-mediated down/up-regulation of FnBPs in these strains.

### Conclusion

From the data presented here, it can be concluded that SaePQ is not required for SDS-mediated SaeS signaling. SDS seems to interfere directly with the SaeS kinase/phosphatase activity and this activity is closely linked to the expression of Eap and the capacity of *S. aureus* to invade host cells. Interestingly a single amino acid exchange (Leu to Pro; L18P) in the putative transmembrane domain of SaeS leads to an opposite output of the SDS mediated signal. This suggests that SaeS activity can be directly modulated by structurally non-complex environmental signals, possibly by altering its kinase/phosphatase activity.
